# Decoding cancer’s camouflage: epithelial-mesenchymal plasticity in resistance to immune checkpoint blockade

**DOI:** 10.20517/cdr.2020.41

**Published:** 2020-10-12

**Authors:** Maria L. Lotsberg, Austin Rayford, Jean Paul Thiery, Giuliana Belleggia, Stacey D’Mello Peters, James B. Lorens, Salem Chouaib, Stephane Terry, Agnete S. T. Engelsen

**Affiliations:** ^1^Centre for Cancer Biomarkers and Department of Biomedicine, University of Bergen, Bergen 5009, Norway.; ^2^BerGenBio ASA, Jonas Lies vei 91, Bergen 5009, Norway.; ^3^INSERM UMR 1186, Integrative Tumour Immunology and Immunotherapy, Gustave Roussy, Fac. de Médecine - Univ. Paris-Sud, Université Paris-Saclay, Villejuif 94805, France.; ^4^Cancer Science Institute of Singapore, National University of Singapore, Singapore, Singapore 117599, Singapore.; ^5^Department of Biochemistry, Yong Loo Lin School of Medicine, National University of Singapore, Singapore, Singapore 119228, Singapore.; ^6^Institute of Molecular and Cell Biology, Agency for Science, Technology and Research, A-STAR, Singapore, Singapore 138673, Singapore.; ^7^Guangzhou Regenerative Medicine and Health, Guangdong Laboratory, Guangzhou 510005, China.; ^8^School of Medicine, Clinical Skills Assessment Program, University of Connecticut, Farmington, CT 06030, USA.; ^9^Thumbay Research Institute of Precision Medicine, Gulf Medical University, Ajman 4184, United Arab Emirates.; ^10^Université Paris-Saclay, INRAE, AgroParisTech, GABI, Jouy-en-Josas 78350, France.; ^*^Equal contribution.

**Keywords:** Epithelial-to-mesenchymal transition, epithelial-mesenchymal plasticity, immune evasion, tumor immune microenvironment, intrinsic and extrinsic mechanisms of resistance to immune checkpoint blockade, therapeutic opportunity

## Abstract

Epithelial-mesenchymal plasticity (EMP) of cancer cells contributes to cancer cell heterogeneity, and it is well established that EMP is a critical determinant of acquired resistance to cancer treatment modalities including radiation therapy, chemotherapy, and targeted therapies. Here, we aimed to explore how EMP contributes to cancer cell camouflage, allowing an ever-changing population of cancer cells to pass under the radar of our immune system and consequently compromise the effect of immune checkpoint blockade therapies. The ultimate clinical benefit of any combination regimen is evidenced by the sum of the drug-induced alterations observed in the variety of cellular populations composing the tumor immune microenvironment. The finely-tuned molecular crosstalk between cancer and immune cells remains to be fully elucidated, particularly for the spectrum of malignant cells along the epithelial to mesenchymal axis. High-dimensional single cell analyses of specimens collected in ongoing clinical studies is becoming a key contributor to our understanding of these interactions. This review will explore to what extent targeting EMP in combination with immune checkpoint inhibition represents a promising therapeutic avenue within the overarching strategy to reactivate a halting cancer-immunity cycle and establish a robust host immune response against cancer cells. Therapeutic strategies currently in clinical development will be discussed.

## Introduction

### The early history of cancer immunotherapy

The history of cancer immunology research dates all the way back to 1863 when Rudolf Virchow observed immune cell infiltration in tumors and hypothesized that sites of chronic inflammation served as a hot bed for cancer development^[1,2]^. Since then, numerous studies have explored the mechanistic link between chronic inflammation and cancer incidence, and tumor-promoting inflammation has been established as a hallmark of cancer^[3-6]^. However, in 1909, Paul Ehrlich postulated that the immune system could also have a protective role against tumorigenesis^[7]^, and this concept was further developed and presented as the “theory of immunosurveillance” by Burnet and Thomas in the 1950’s^[8-11]^. Despite the fact that these early studies established a link between cancer and the immune system, the importance of the immune system in cancer progression and the therapeutic possibilities for cancer treatment remained largely neglected for nearly half a century. In fact, the theory of immunosurveillance was considered controversial until an important scientific discovery was published in *Nature* by Shankaran *et al*.^[12]^ in 2001. The experimental evidence presented in this paper unambiguously showed that the immune system can, and often does, prevent tumors from developing, and that the immune status of mice is a critical determinant of their susceptibility to tumor development induced by chemical carcinogens^[12,13]^.

A malignant cell may reveal its identity and alert the immune system in various ways. During malignant progression, cancer cells accumulate genetic mutations, which in turn may result in the expression of tumor-specific antigens, also known as neo-antigens. In addition, epigenetic alterations may also deregulate the expression of tumor-associated antigens such as cancer embryonic antigens or cancer testis antigens. Both tumor-specific and tumor-associated antigens can be recognized as foreign by the immune system and initiate immune cell-mediated clearing of the malignant cell population^[14,15]^. However, the strong selection pressure towards cancer cells that continuously expose tumor antigens and markers of cellular distress enforces a continuous interplay between cancer cells and immune cells, referred to as the process of “immunoediting”^[9,13]^. As a consequence of this constant selection pressure, malignant cells evolve to escape the immune system, and thus the concept of immunoediting could in part explain what was previously considered a controversy: the immune system may contribute to tumor suppression as well as tumor promotion^[13]^.

Conceptually, the process of immunoediting consists of three sequential phases^[9]^. In the first phase, the innate and adaptive arms of the immune system work in concert to attack and eliminate the malignant cells (elimination phase). In the second phase, the adaptive arm of the immune system keeps the cancer under control by eliminating some cancer cells while others survive and proliferate, resulting in an equilibrium between dying and proliferating cells in the malignant tissue (equilibrium phase). In this phase, the cells that are still actively dividing will give rise to a population of more or less immunogenic cells, and clonal expansion of less immunogenic cancer cells results in the third phase of the immunoediting process, wherein the tumor escapes from the immune response (escape phase). It is believed that malignancies can remain dormant and asymptomatic in the equilibrium phase of immunoediting for a prolonged period of time, in some cases even an entire lifetime, unless they can eventually “escape” the immune pressure and manifest as symptomatic and clinically detectable cancers. This notion is supported by autopsy studies that have revealed the prevalence of well-differentiated indolent malignancies or “pseudo cancers” of the thyroid, prostate and mammary glands^[16]^. The onset of the escape phase may be a result of alterations in the cancer cells themselves or through alterations in the tumor immune microenvironment (TIME), leading to an immunosuppressive state^[9,13]^. As all clinically detectable cancers have successfully escaped the immune response at least to some degree, the process of immunoediting represents a non-linear model where the evolution of the malignant cells and the TIME are mutually dependent on each other. The goal of cancer immunotherapy is to shift the equilibrium of cancer immunoediting from tumor tolerance towards eradication by modulating the crosstalk between malignant cells and the TIME.

### The immune-oncology revolution

William Coley was the first to demonstrate that immune cells can kill cancer cells in patients. Already in 1893, he treated cancer patients with a mixture of bacterial toxins (Coley’s toxins) in a successful attempt to activate the immune system to attack cancers^[17,18]^. Another approach to boost the immune system to fight cancer has been used for decades in the clinical management of early stage high-risk non-muscle-invasive bladder cancer, where Bacillus Calmette-Guerin, a vaccine originally intended for immunization against *M. tuberculosis*, is applied as an intravesical immunotherapy to induce a localized immune response^[19,20]^. However, immunotherapy did not emerge as a revolutionary cancer therapy for multiple cancer types until the recent development of immune checkpoint blockade (ICB) therapy. The concept of ICB is to utilize antibodies to block the receptor-ligand interactions of molecules that serve as negative regulators of the immune system, such as cytotoxic T lymphocyte-associated protein 4 (CTLA-4) and programmed cell death protein 1 (PD-1). Work by Allison and colleagues showed that CTLA-4 functions as an immune checkpoint or “off-switch” by binding CD80/CD86 with greater affinity than the stimulatory “on-switch” CD28, thereby inhibiting activation and proliferation of T cells^[21,22]^. Ipilimumab (Yervoy®, Bristol-Myers Squibb, New York City, NY, US) is a humanized antibody that blocks CTLA-4, thereby reactivating the immune system. The clinical trials leading to US Food and Drug Administration (FDA) approval of ipilimumab in 2011 received enormous attention, as it was the first cancer treatment *ever* to demonstrate an increased overall survival in metastatic melanoma patients^[23]^.

The interaction between the PD-1 receptor and its inhibitory ligand programmed death-ligand 1 (PD-L1) is another example of an inhibitory checkpoint modulating the duration and amplitude of the immune response against cancer cells^[24-26]^. Interactions between PD-1 and PD-L1 cause inhibition of T cell receptor (TCR)-mediated lymphocyte proliferation and cytokine secretion^[26]^. In a physiological context, PD-L1 plays an important role in protecting host cells from immune-mediated tissue damage post-infection and preventing autoimmune disorders. PD-L1 is frequently overexpressed by malignant cells, allowing cancers to escape the adaptive immune system^[27]^. Numerous studies have reported that increased expression of PD-L1 in tumors is associated with poor prognosis^[28-31]^. Antibodies blocking the PD-1/PD-L1 axis have been shown to induce durable clinical responses in numerous cancer types including malignant melanoma^[22,23,32]^, non-small cell lung cancer (NSCLC)^[33,34]^, urothelial carcinoma^[35,36]^, head and neck squamous cell carcinoma, Hodgkin’s lymphoma and renal cell carcinoma (RCC)^[32-34,37-39]^. This has led to FDA approval of several immune checkpoint inhibitors including the fully humanized monoclonal PD-L1-targeting antibodies atezolizumab (Tecentriq®, Genentech/Roche, South San Francisco, CA, US) and durvalumab (Imfinzi®, AstraZeneca, Cambridge, UK), as well as monoclonal antibodies targeting PD-1; namely pembrolizumab (Keytruda®, Merck Sharp & Dohme, Kenilworth, New Jersey, US) and nivolumab (Opdivo®, Bristol-Myers Squibb, New York City, NY, US)^[35,36,40,41]^.

### The remaining challenge

ICB truly represents a paradigm shift in cancer treatment^[42]^; not only do some patients experience profound clinical benefit from the treatment, but also it has been shown to induce durable responses for a subset of patients with aggressive malignancies^[42,43]^. Unfortunately, some of the most common cancers such as breast, prostate and colon cancer have shown poor responses to ICB^[37,44]^. Furthermore, even within the subtypes of cancers for which ICB therapy is indicated, heterogeneous responses have been shown, and a majority of the patients do not benefit from ICB therapy^[42]^. Furthermore, heterogeneous responses have been observed between multiple cancer lesions within the same patient^[44]^. The European Organisation for Research and Treatment of Cancer 18071 trial reported that more than half of high-risk patients with stage III melanoma who received adjuvant ipilimumab relapsed with a median recurrence-free survival of 26.1 months^[45]^. The KEYNOTE-001 trial reported that one in four patients with metastatic melanoma who achieved an initial objective response to pembrolizumab subsequently experienced disease progression^[45]^. However, the reported outcomes of the efficacy of pembrolizumab treatment either alone or in combination with other treatments for advanced/metastatic melanoma varies considerably in the multiple studies performed, and a recent meta-analysis including 25 research articles with a total of 2,909 patients reported an overall response rate of only 34% (progression-free survival: 5.7 months, overall survival 20.3 months)^[46]^.

Multiple mechanisms have been proposed to explain why only a subset of tumors respond to ICB, yet an urgent lack of reliable predictive biomarkers remains^[37,42,47]^. High cancer cell expression of immune checkpoint ligands, high tumor mutational burden (TMB) and a favorable immune cell infiltration in the TIME, i.e., immune cell inflamed “hot” as opposed to immune cell excluded “cold” tumors, have all been associated with a more favorable response to ICB^[37,48,49]^. The best predictive biomarkers identified so far include the TMB and neoantigen landscape of the tumor; however the adoption and harmonization of these methods in the clinic remain in their infancy^[15,50,51]^. For lack of better alternatives, PD-L1 expression is currently being used as a predictive biomarker for immune checkpoint inhibitors targeting the PD-1/PD-L1 axis in malignant melanoma, NSCLC and RCC, but the usefulness of PD-L1 expression as a predictor of response to ICB remains a continuous matter of debate^[42,52]^. Thus, extensive efforts are currently being made to identify better predictive biomarkers and explore rational combinational therapies, with the ultimate aim to increase initial ICB response rates while also preventing or reversing the emergence of resistance^[49,53]^.

In this review, we aim to explore how epithelial-mesenchymal plasticity (EMP), i.e., the acquired ability of epithelial cells to transition between epithelial and mesenchymal phenotypes, might contribute to cancer cell camouflage, enabling an ever-changing population of cancer cells to escape the immune system. Targeting EMP represents a promising therapeutic opportunity in combination with immune checkpoint inhibitors. Here, we aim to elucidate how EMP affects the various steps of the cancer-immunity cycle and to highlight some of the clinical evidence and molecular mechanisms supporting the hypothesis that targeting EMP represents a promising therapeutic avenue within the overarching strategy to reactivate a halting cancer-immunity cycle and establish a robust host immune response against cancer cells.

## Main text

### Epithelial-mesenchymal transition and plasticity

The majority of human cancers are of epithelial origin^[54]^. As opposed to mesenchymal cells, which are characterized by their spindle-shaped fibroblast-like morphology and loose connection to surrounding cells, epithelial cells exhibit highly organized apicobasal polarity and junctional complexes ensuring stable intercellular adhesion and controlling transepithelial permeability. The process of epithelial-to-mesenchymal transition (EMT) was first described in embryonic development^[55-57]^, but was later associated with physiological and pathological processes occurring in adults such as wound healing, in the progression of carcinoma and indirectly in tissue fibrosis^[58-63]^. EMT is a cellular process where epithelial cells lose their cell polarity and tight cell-cell adhesions, acquiring a fibroblast-like morphology and cytoarchitecture as well as the ability to remodel the extracellular matrix (ECM) and alter their cellular behavior to gain more migratory and invasive properties^[55,62,64]^. Considerable remodeling of the cytoskeleton accompanies the loss of epithelial cell polarity and confers new mechanobiological properties favoring migration and tissue invasion^[62,63]^. At the molecular level, EMT is characterized by downregulation of epithelial markers (e.g., E-cadherin and EpCAM), and upregulation of mesenchymal markers (e.g., N-cadherin and vimentin). Mesenchymal cells also exhibit increased expression of matrix metalloproteinases which are required for their invasive properties^[65,66]^. The most commonly recognized EMT transcription factors (TFs) include Snail (SNAI1), Slug (SNAI2), zinc finger E-box binding homeobox 1 and 2 (ZEB1 and ZEB2), and Twist family BHLH transcription factor 1^[62,67]^. Since multiple TFs may activate EMT, different combinations of TFs may be responsible, at least in part, for the heterogeneity that stems from different versions of the EMT program^[60]^. Of note, the EMT International Association suggests that EMT should not be assessed solely on the basis of EMT-TF expression or that of a small number of molecular markers, but rather the primary criteria for defining EMT should consist of measurable alterations in cellular characteristics together with a set of molecular markers^[62]^.

Importantly, EMT is not an irreversible linear process, and the reverse process, mesenchymal-to-epithelial transition (MET), describes the transition from the mesenchymal (M) to the epithelial (E) state^[68]^. Although EMT was initially believed to be a binary process, it is now well documented that this is indeed a dynamic process and intermediate-state cells also exist^[61,69]^. Cells can thus undergo partial EMT or partial MET to obtain intermediate E/M states with a cellular phenotype that is neither completely epithelial nor completely mesenchymal and harbors attributes characteristic of both states^[62,69]^. Rather than acting as an “on/off” switch between the E and M states, the process of EMT is therefore considered to generate a continuum of cell states along an “epithelial-mesenchymal axis”, also frequently referred to as an “EMT scale” or “EMT spectrum”^[60,70]^. Tan and colleagues have developed a universal and quantitative gene expression-based EMT scoring system used to establish and evaluate an EMT spectrum across different cancer types^[69]^. Recently, the term “epithelial-mesenchymal plasticity” (EMP) has been increasingly recognized, referring to the ability of cells to adopt mixed E/M features and to switch between various states along the epithelial-mesenchymal spectrum^[62]^. Although carcinoma cells may hijack this conserved developmental program, its physiological role in maintaining homeostasis is highly regulated both spatially and temporally, while the pathological EMT program detected in cancers, on the other hand, appears to be a mainly stochastic and time-independent process^[65,66,71]^. Of note, non-epithelial cancers such as melanoma have also been shown to undergo EMT-like programs leading to similar phenotypic changes induced by EMT-related genes, and serve as a model system for studying metastasis and therapy resistance^[72-74]^. Phenotypic switching in melanoma involves re-organization of the cytoskeleton, cell membrane and cellular adhesions, as well as alterations in common EMP-related phenotypic markers including gain of E-cadherin and loss of N-cadherin expression^[72]^.

Under pathological conditions, epithelial cells are believed to undergo EMT, but they rarely or perhaps never undergo the full EMT transition to obtain a completely mesenchymal phenotype, suggesting that partial EMT represents the norm rather than the exception^[60,62]^. Evidence also indicates that these intermediate E/M phenotypes have the greatest malignant and metastatic potential^[75-77]^. Carcinoma cells can also temporarily transition to a more plastic state to metastasize or overcome selection pressures from therapy and subsequently revert to an epithelial-like state^[75]^. Expression of EMT-TFs is also frequently accompanied by features of stemness. Retention of stemness has been linked to EMT and in particular to a stemness window around intermediate E/M states^[61]^. Like EMP, the acquisition of stem cell traits in cancer cells is not considered a fixed trait; rather it represents a hallmark of cancer within a spatial and temporal window in cancer progression^[61]^. Accumulating evidence suggests that morphological phenotype and stemness can be regulated independently in malignant cells, but that a stemness window along the EMT axis may predispose malignant cells for acquisition of stem cell traits^[61]^.

#### EMP as a mediator of resistance to cancer therapies

Even for the most effective cancer therapies, be it chemotherapy, targeted therapies, radiotherapy or immunotherapy, persisting cancer cells that survive treatment are believed to constitute a reservoir of slow-cycling cells that eventually may acquire irreversible genetic mutations or epigenetic alterations causing therapy resistance and relapse^[78-80]^. Acquired therapy resistance is one of the major obstacles to achieving durable remission, and thus major efforts have been made to understand the mechanisms of drug resistance against targeted therapies as well as more conventional cancer treatment regimens. Historically, the focus of this research has been to uncover genetic mutations responsible for acquired drug resistance, such as secondary mutations in the target gene, causing impaired drug binding, or mutations causing activation of downstream signaling or alternative survival pathways^[78,81]^. However, recent attempts to characterize resistance mechanisms to targeted therapies, such as that of third-generation anaplastic lymphoma kinase (ALK) inhibitor loratinib resistance by next-generation sequencing and phenotypic analysis of longitudinal tumor samples, have highlighted EMT as a mediator of resistance in cases where a specific mutation affecting drug binding could not be detected^[82]^. Furthermore, the EMT-mediated loratinib resistance found in these patient-derived cell lines could be overcome by combined SRC and ALK inhibition^[82]^. EMP has been shown to be widely associated with therapy resistance against both cytotoxic and targeted therapy in multiple cancer types, and increasing evidence also shows that EMP contributes to a multidrug resistance phenotype^[58,60,71,83]^. However, the exact mechanism by which EMP contributes to drug resistance in these various contexts remains to be fully elucidated. Proposed mechanisms include reduced levels of pro-apoptotic proteins or increased drug efflux, which may be part of a broader association between EMT-mediated cancer cell dedifferentiation, acquisition of stem cell traits, and drug resistance^[78,83-87]^. Although it has been acknowledged for quite some time that dynamic chromatin modifications may be an independent route to drug resistance in cancer cells and thus embodies a promising drug target in combination with various drugs including ICB, it was not until the recent FDA approval of tazemetostat (Trade name: Tazverik, developer: Epizyme), a first-in-class small molecule inhibitor of enhancer of zeste homolog 2 (EZH2), that this hypothesis has been feasible for clinical testing. Tazemetostat was FDA approved as a monotherapy in January 2020 for patients with histologically confirmed, metastatic or locally advanced epithelioid sarcoma with INI1 loss, and was thus the first “epigenetic” drug approved for solid cancers. EZH2 is considered a master regulator of EMT through orchestrating the regulation of the H3K27me3 epigenetic mark, and in pancreatic cancer it has been shown to regulate EMT through miRNA 139-5p. Tazemetostat is currently in phase 2 clinical trials in combination with the PD-1 inhibitor Tecentriq^TM^ (atezolizumab) in patients with relapsed or refractory diffuse large B-cell lymphoma (DLBCL), and thus future combination studies will need to address whether EZH2 inhibitors or other epigenetic drugs can reduce EMT and increase response to ICB in pancreatic cancer or other solid cancers.

Novel technologies leveraging single cell analysis have only just begun to provide a better mechanistic understanding of the processes at play. In several recent studies *in vitro*, drug-tolerant persister cells, sometimes referred to as “jackpot” cells, have been observed in cancer cell cultures from a variety of tumor types upon treatment^[78,80,88-91]^. From single-cell sequencing endeavors, these studies have shown that many cancers, including melanoma, display a profound transcriptional variety at the single cell level. Through single-cell gene expression analyses of melanoma cultures, Shaffer and colleagues were able to demonstrate that non-heritable resistance to the V600E mutated BRAF inhibitor vemurafenib (Zelboraf, Plexxikon and Genentech) was established from a very rare (1 per 50-500 cells) subpopulation of pre-existing pre-resistant cells. These cells transiently express a semi-coordinated set of well-known plasticity and resistance markers, including epidermal growth factor receptor (EGFR), platelet derived growth factor receptor beta, nerve growth factor receptor, fibroblast growth factor receptor 1, Wnt family member 5A (WNT5A), JUN and the receptor tyrosine kinase AXL^[91]^. Treatment with vemurafenib induced a stepwise reprogramming of the pre-resistant cells into a stable resistant phenotype, which was not affected by drug holidays^[91]^.

AXL expression in melanoma has been associated with resistance mechanisms mediated by mitogen-activated protein kinase (MAPK)/extracellular signal-regulated kinase (ERK) signaling^[92-94]^. Tirosh and colleagues provided strong evidence for the involvement of AXL as well as AXL-related genes (AXL signature) in acquired melanoma resistance to both vemurafenib and the MEK inhibitor trametinib (Mekinist, Novartis, Basel, Switzerland), and also found that the proportion of a similar population of “jackpot” cells as well as the abundance of plasticity gene transcripts significantly increased after resistance was acquired *in vitro*, while being selected against more differentiated populations expressing melanocyte-inducing transcription factor (MITF) and SRY-box transcription factor 10^[95]^. Taken together, the accumulating evidence for the role of rare “jackpot” cells in acquired therapy resistance supports the hypothesis that intervention with cytotoxic or targeted therapies and immunotherapies displaying initial efficacy in tumor eradication may also act as selection pressures, predisposing tumors to immune evasion through the acquisition of plasticity phenotypes and priming cells for immune resistance. The adoption of distinct phenotypes through the process of EMT may provide cells with properties adaptive to changes in the TIME, and thus act as drivers towards a phenotypic state independent of the drug-targeted pathways^[78]^.

#### The tumor immune microenvironment as a regulator of epithelial-mesenchymal plasticity

The tumor microenvironment is a key regulator of EMP, which may be induced by combinations of multiple microenvironmental factors including hypoxia, pH, ECM composition, tensile forces and the presence of soluble factors such as interferons, inflammatory cytokines and growth factors^[61,65,66,86,96-98]^. Hypoxia occurs in most actively growing solid tumors and is an important EMT inducer. Accumulating evidence also suggests that hypoxic stress is linked to therapy resistance as well as regulation of tumor immunogenicity and both tumor and immune plasticity^[99,100]^. Hypoxic areas of growing tumors have been shown to attract immunosuppressive cells such as myeloid-derived suppressor cells (MDSCs), tumor-associated macrophages (TAMs) and regulatory T cells (Tregs)^[101]^, and hypoxia has also been shown to upregulate PD-L1 on MDSCs^[102,103]^. Targeting hypoxia has thus been suggested to improve the efficacy of cancer immunotherapy^[102,104,105]^. Remodeling of ECM through secretion of specific matrix MMPs and new ECM components, namely collagen type 1, has been shown to induce EMT through both mechanotransduction and direct membrane receptor signaling through integrins and DDR1/2^[106-108]^. Cancer-associated fibroblasts (CAFs), in addition to their role in ECM remodeling, serve as one of the primary sources of key EMT-inducing growth factors including hepatocyte growth factor (HGF), fibroblast growth factor, interleukin (IL)-6 and transforming growth factor-beta (TGF-β); however, molecular profiling and ablation of heterogeneous CAF subsets in various tumor models have shown conflicting and context-dependent effects on the efficacy of different therapies^[109]^. Thus, for CAFs and other cellular components of the TIME, the continuum of cells along the differentiation/polarization axis is far more complex than previously anticipated and remains to be explored further by high-dimensional analyses.

Multiple studies have demonstrated that the TIME strongly contributes to the induction of the EMT program and subsequent tumor progression^[66]^. Cells of the TIME that are involved in regulating EMP include TAMs, MDSCs, neutrophils and natural killer (NK) cells^[110,111]^. TAMs have a major role in orchestrating cancer-related inflammation, and pro-inflammatory macrophages have been shown to induce EMT at the invasive margin of the tumor through inflammatory cytokine tumor necrosis factor-α (TNF-α)-mediated stabilization of the transcription factor SNAI1 via nuclear factor-κB (NF-κB) pathway activation, while knockdown of SNAI1 suppressed inflammation-mediated breast cancer metastasis, suggesting a mechanism for EMT as a regulator of inflammation-induced metastasis^[112]^. Macrophages have also been shown to induce EMT in intratumoral cancer cells via TGF-β secretion and activation of the β-catenin pathway^[113]^. In a murine melanoma model, MDSCs were shown to infiltrate the tumor and induce EMT *in vivo*^[114]^. Functional *in vitro* assays with purified MDSCs revealed that the TGF-β, epidermal growth factor (EGF) and HGF signaling pathways were all involved in MDSC-induced EMT activation of cancer cells^[114]^. In a similar model, HGF secretion by T cell-inflamed TIMEs was shown to mobilize and recruit neutrophils through binding and activation of c-MET, wherein they gained immunosuppressive properties^[115]^. Neutrophils are also frequently enriched at the invasive margin of gastric cancers, and in a recent study, tumor-associated neutrophils were shown by *in vitro* co-culture experiments to promote EMT in gastric cancer cells via IL-17α signaling^[116]^. Although the mechanisms are still unclear, NK cells may increase the malignancy of melanoma cells by inducing an EMT-like switch^[110]^.

### The role of EMP in the cancer-immunity cycle

The finely tuned, step-wise process by which the immune system can efficiently recognize and eradicate cancer cells was first conceptualized by Chen and Mellman and is now widely known as “the cancer-immunity cycle”^[117]^. In the first step of the cancer-immunity cycle, cancer antigens are recognized and captured by professional antigen-presenting cells (APCs), such as dendritic cells (DCs)^[117]^. Alarmins or damage-associated molecular patterns (DAMPs) released by dying tumor cells may aid in the attraction of APCs to the tumor bed^[118,119]^. This initiation of the cancer-immunity cycle is dependent on the APCs capturing both cancer specific antigens expressed by the cancer cells (immunogenicity) as well as signals that specifically evoke an immune response (adjuvanticity)^[117,120]^. Toll-like receptors (TLRs) are expressed on DCs and are key mediators bridging innate and adaptive immunity once stimulated by foreign pathogen associated molecular patterns (PAMPs) or DAMPs. TLR activation results in the secretion of pro-inflammatory cytokines including IL-6, IL-12, TNF-α, and type I interferons, which in turn serve to recruit immune effector cells. The APCs from step one of the cancer-immunity cycle travel from the tumor microenvironment to the lymph node where they present captured cancer related antigens to T cells (step 2). T cells can then be activated and programmed to specifically react to these antigens (step 3). Activated cytotoxic T lymphocytes (CTLs) then travel back to the tumor site (step 4) where they are then able to infiltrate the tumor (step 5), recognize (step 6) and kill (step 7) the antigen-presenting cancer cells^[117]^. All these steps need to be intact for the cancer-immunity cycle to be successfully completed.

**Figure 1:**
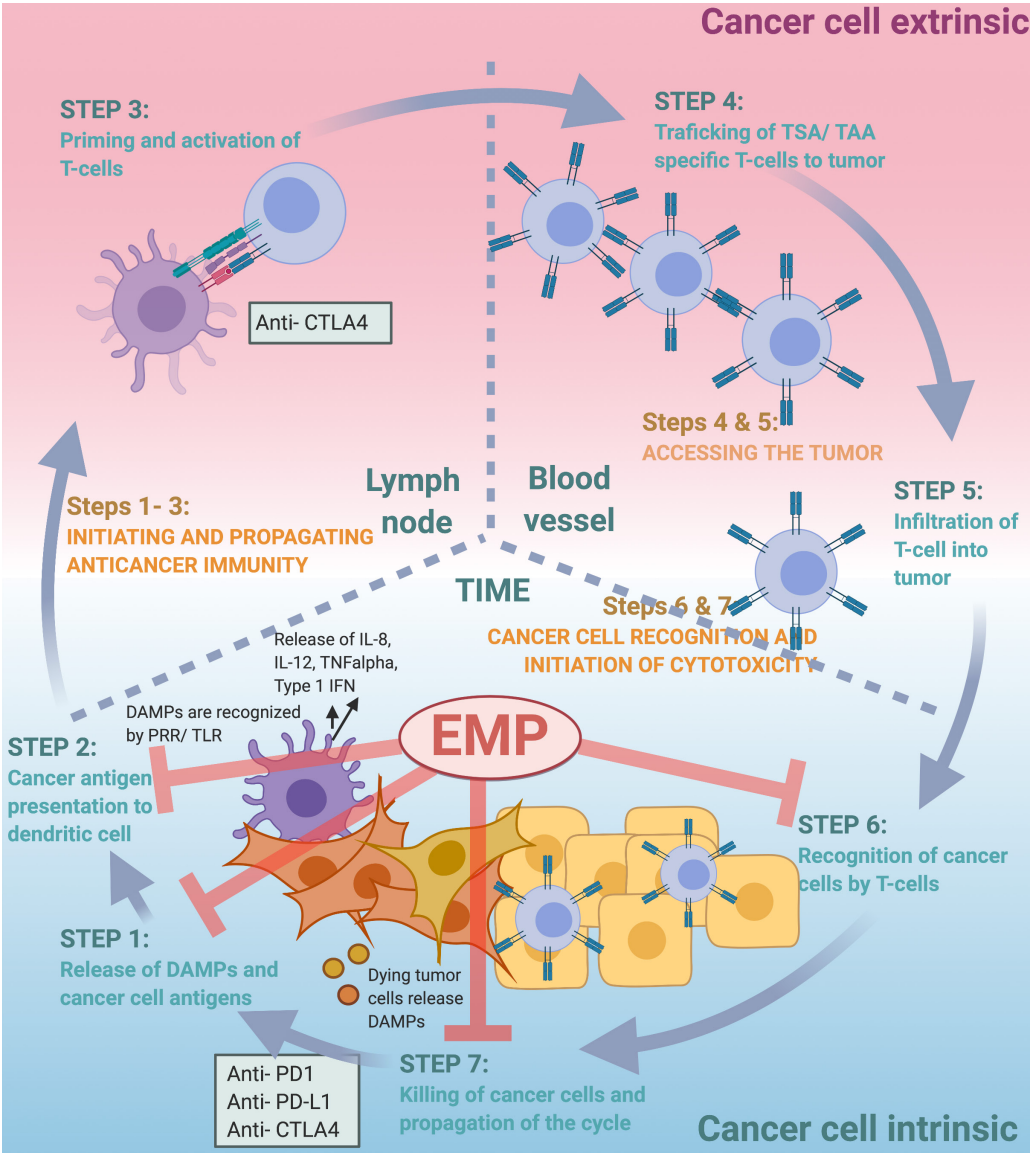
EMP affects various steps of the cancer-immunity cycle. The therapeutic rationale for targeting EMP in combination with ICB is based on the fact that EMP affects multiple steps of the cancer-immunity cycle described by Chen and Mellman^[117]^. Briefly, targeting EMP can induce an increased release of DAMPs serving as an adjuvant during the release of cancer cell antigens (STEP 1). DAMPs are further recognized by pattern recognition receptors (PRRs) including TLRs. EMP targeting can induce an M1 to M2 polarization of macrophages and an activation of APCs, and thus aid in cancer antigen presentation (STEP 2). Targeting EMP and the EMP-associated immunosuppressive tumor immune microenvironment (TIME) can enable the infiltration of educated T cells into the cancer (STEP 5). EMP is associated with reduced recognition (STEP 6) and immune effector cell-mediated killing (STEP 7) of cancer cells, and targeting EMP can therefore induce increased effector cell-mediated lysis of cancer cells and propagation of the cycle. Adapted courtesy of Chen and Mellman^[117]^. EMP: epithelial-mesenchymal plasticity; ICB: immune checkpoint blockade; DAMPs: damage-associated molecular patterns; APCs: antigen-presenting cells

As discussed below, EMT may affect several steps of the cancer-immunity cycle and thus hinder progression in this finely-tuned cycle in multiple ways [Figure 1]. One of the remaining challenges will be to identify the “Achilles heel” of individual cancer cases and tailor the treatment to initiate a robust cancer-immunity cycle. We suggest that targeting cancer EMP may represent a unique opportunity to bolster several steps of the cancer-immunity cycle. Although the molecular mechanisms of EMP affecting the various steps of the cancer-immunity cycle are still largely obscure, targeting EMP represents a promising therapeutic addition within the overarching strategy of personalized cancer immunotherapy to re-activate a halting cancer-immunity cycle and re-establish a robust host immune response against the cancer cells^[53,121]^.

Using hypoxia-inducible factor 1-alpha (HIF1 alpha) and AXL inhibitors as examples, the rationale behind clinical trials combining ICB and mediators of epithelial phenotypic plasticity are based on the effects of these drugs on multiple steps of the cycle. First, cancer treatments that induce immunogenic cell death (ICD) have been shown to contribute to anticancer immune attack, mediated by the exposure of DAMPs. Hypoxia is a strong microenvironmental factor that supports coordinated induction of EMT and also AXL expression and autophagy. The HIF1 alpha inhibitor PX-478 was also recently shown to enhance gemcitabine-induced immune responses and eliminate pancreatic ductal adenocarcinoma (PDAC) cells through induction of ICD^[122]^. EMT correlates with autophagy induction, and pre-mortem stress-related autophagy is also linked to the release of DAMPs and induction of ICD^[53,123]^. Thus, the impact of targeting autophagy to increase the efficacy of immune checkpoint inhibition is expected to be seen particularly in cancers of an immune cold or immune-excluded immunophenotype^[49]^. We have recently shown that the small molecule AXL inhibitor bemcentinib abrogates the autophagic flux and is a potent inducer of ICD; this is of particular interest as it implicates a favorable adjuvant effect and alteration of the TIME upon AXL inhibition in immunogenic but immunosuppressed tumors^[53,124]^. In this context, it is important to note that the increased danger signaling *per se* is not sufficient to compensate for the intrinsically low immunogenicity of some tumors, and that the effect of autophagy-dependent danger signaling is solely expected to warm up immune cold tumors and elicit a strong immune response in immunogenic tumors^[125]^. Increased immunoadjuvanticity, that is, the recruitment of professional APCs to educate the naïve T lymphocyte population, is critical to maximize the potential therapeutic benefit of ICB. Furthermore, AXL has been shown to suppress antigen presentation by downregulating major histocompatibility complex I (MHC-I), and genetic ablation of AXL in the experimental PyMT-induced tumor model was shown to induce MHC-I expression to even higher levels compared to IFN-γ-induced parental cells^[126]^. AXL inhibition enhances cytokine release and increases CD8+ T cell response in syngeneic models, resulting in an elevated cytotoxic T cell-dependent antitumor immune response after radiation, which could be further enhanced by ICB^[126]^.

Carcinoma subclones with pronounced mesenchymal phenotypes emerging from sustained hypoxic stress of primary NSCLC cells in culture were shown to be less susceptible to NK and CTL cell-mediated lysis compared to subclones with a more epithelial phenotype^[96]^. This difference was associated with reduced expression of intercellular adhesion molecule 1 (ICAM1), UL16 binding protein 1 (ULBP1), and MHC-I as well as increased TGF-β expression in mesenchymal subclones^[96]^. It was shown that expression of AXL in the mesenchymal NSCLC clones was correlated with an increased cancer cell intrinsic resistance to immune cell-mediated killing by NK cells as well as autologous CTLs while AXL targeting via small molecule inhibition potently sensitized mesenchymal lung cancer cells to cytotoxic lymphocyte-mediated killing^[127]^. These findings raise the intriguing possibility that phenotypic plasticity may enable tumor cells to circumvent NK- and CTL-mediated cell killing. This has also been attributed to the associated cytoskeletal remodeling in tumor cells, interfering with immune synapse formation^[96,128,129]^. This process may provide a rationale to test EMP-targeting approaches to enhance antitumor immunotherapy^[95,130,131]^.

The EMT-TF SNAI1 has been shown to have a strong impact on the cancer-immunity cycle. In human mammary carcinoma MCF7 cells, Akalay *et al*.^[132]^ observed that overexpression of exogenous SNAI1 correlated with increased mesenchymal and stemness traits in these cells while reducing their susceptibility to CTL-mediated lysis. This was in part explained by reduced MHC expression in the SNAI1-transfected MCF7 derivatives and concomitant reduced activity of TCR signaling at the site of cell-cell contact (immunological synapse). Similar observations were reported using another MCF-7 derivative displaying hyperactive TGF-β signaling. Likewise, SNAI1 overexpression in murine B16 melanoma cells resulted in inhibition of CTL lysis activity, inhibition of DC maturation and expansion of suppressive Treg-like CD4+ Foxp3+ cells in a mechanism involving thrombospondin (TSP1) and TGF-β secretion^[130]^. Using PyMT cells, a murine transplantable model of breast cancer carcinoma, Dongre and colleagues showed that tumors arising with a more mesenchymal phenotype and high expression of SNAI1 expressed more PD-L1 and less MHC-I and had less cytotoxic T cells, but were enriched for immunosuppressive components including M2 macrophages and Tregs^[133]^. The link between EMP and the presence of immunosuppressive macrophages seems to be critical. Although the mechanisms are still unclear, it is tempting to speculate the involvement of immunosuppressive chemoattractants (e.g., TGF-β) in the recruitment of macrophages to the TIME. Another interesting observation made by Dongre and colleagues was that NK cells infiltrate mesenchymal tumors more effectively compared to the epithelial tumors, which can be explained by downregulation of MHC-I in the more mesenchymal tumors^[133]^. However, despite this NK enrichment, the mesenchymal tumors rapidly grow in this model suggesting intrinsic or extrinsic resistance as well as possible cooperative effects.

Converging preclinical evidence also suggests that tumors with a more EMP/mesenchymal phenotype are less responsive to ICB. SNAI1+ melanoma tumors were unresponsive to immunotherapy, but targeting SNAI1 with siRNA or anti-TSP1 monoclonal antibody could inhibit tumor progression and induce systemic immune responses^[130]^. In ZEB1/miR-200-driven NSCLC models as well as in SNAI1-driven breast cancer models, EMP was associated with PD-L1 expression^[134]^. Targeting PD-L1 in NSCLC efficiently prevented tumor growth and metastasis. In PyMT breast cancer models, epithelial tumors were susceptible to elimination by anti-CTLA-4 immunotherapy, whereas corresponding mesenchymal tumors were refractory to such treatment^[133]^. By injecting E/M cells at various ratios, these investigators also elegantly demonstrated that in the case of mixed epithelial-mesenchymal tumors, even the presence of rare mesenchymal carcinoma cells could protect the epithelial counterpart from anti-CTLA-4 treatment by promoting the recruitment of immunosuppressive cells including M2 macrophages and Tregs^[133]^.

### EMP as a mediator of primary and acquired ICB resistance

Sharma and colleagues have described three different categories of resistance against immunotherapy: primary, adaptive and acquired resistance^[37]^. Primary, or innate, resistance to immunotherapy occurs when the cancer does not have an initial response (i.e., it is refractory) to the therapy. Adaptive resistance occurs when the tumor is initially recognized by the immune system, but the tumor adapts to escape immune attack. Given the evolving nature of the interaction between immune cells and cancer cells in this process, adaptive resistance can manifest clinically as either primary resistance, mixed responses or acquired resistance. Thus, only clinical data describing the role of EMP in primary and acquired resistance will be discussed here. Acquired resistance describes the clinical scenario in which the cancer initially responded to immunotherapy but recurs and progresses after a period of time. However, as Sharma and colleagues emphasize in their review, the immune response is dynamic and can evolve for each patient and tumor, and could also be affected by environmental and genetic factors as well as by the cancer treatment^[37]^. Accordingly, it is important to note that the process of immunoediting mentioned above is anything but a static process, as novel rounds of therapy and subsequent immunoediting affect the composition of the TIME through clonal selection acting on pre-existing cancer cell populations or *de novo* acquisition of resistant traits.

#### EMP in primary ICB resistance

The hypothesis that epithelial phenotypic plasticity could be a key determinant of the outcome of immune checkpoint inhibition in cancer is fueled by pioneer transcriptional data^[135]^. Hugo and colleagues performed exome and transcriptomic sequencing of melanoma pre-treatment samples, and a common gene signature was shown to characterize tumors that were non-responsive to anti-PD-1 therapy^[135]^. This innate PD-1 resistance (IPRES) gene signature encapsulates the upregulation of EMT transcription factors, immunosuppressive cytokines, and pro-angiogenic factors^[135]^. The IPRES signature, enriched in non-responding patients, overlaps with signatures for wound-healing, angiogenesis, ECM remodeling, cell adhesion, monocyte/macrophage chemotaxis and resistance to MAPK pathway inhibition^[135]^. Data from The Cancer Genome Atlas (TCGA), PROSPECT, and BATTLE-1 showed that inflammatory changes in the tumor microenvironment were strongly associated with induction of EMT signatures in lung adenocarcinoma, which in turn correlated with upregulation of multiple suppressive immune checkpoint receptors or their ligands, including B7-H3, CTLA-4, PD-1, PD-L1, PD-L2, B and T lymphocyte attenuator, and T cell immunoglobulin and mucin-domain containing-3 (TIM-3)^[45]^. Taken together, these data suggest that attenuating the mechanics underlying the IPRES signature may have the potential to enhance anti-PD-1 responses in melanoma and other cancers. Intriguingly, the receptor tyrosine kinase AXL, whose upregulation is associated with a reversible plasticity cell state^[94]^ and is part of the “jackpot” signature of pre-existing pre-resistant cells^[91]^, was found to be a component of the IPRES signature among 532 upregulated genes including several EMT and EMP mediators such as *EGFR, NGF, CDH11, EPHA3, HEY1, HEY* (NOTCH effectors), *TWIST2, ID3, ID1*, many interleukins including immunosuppressive *IL10, ITGA5/8; LOX, LOXL2, MMP1, MMP2, MMP3, MMP9, NRP1, THBS1* and *THBS2, NUMBL* (plasticity factor), *ROR2, RORB, RUNX2, SEMA3A SERPINE1 VEGFA, VEGFC* and Wnt genes *WNT11 WNT2 WNT5A WNT7B* and *DKK3.* Among the 161 genes downregulated in the IPRES signature are *HLA-A* and *E-cadherin (CDH1)*^[135]^.

In line with these findings from melanoma, Thompson and colleagues recently observed that NSCLC tumors displaying a more mesenchymal phenotype showed reduced clinical responses to ICB^[136]^. In this study, an EMT/inflammation-based signature had clinical utility in predicting clinical response^[136]^, supporting the view that the EMP signature is an adverse predictor of ICB response across tumor types.

Furthermore, in urothelial cancer patients with T cell-infiltrated tumors, a higher EMT/stroma-related gene expression signature was associated with poor response and disease progression under anti-PD-1 therapy^[137]^. This study suggested that stromal elements are the major source of immune resistance in this setting, thus providing a rationale for co-targeting PD-1 and the stromal elements mediating resistance^[137]^.

#### EMP in acquired resistance to ICB

Potential mechanisms of acquired resistance to immunotherapy include loss of T-cell function, downregulation of tumor-associated antigen presentation, and acquisition of mutations that enable immune escape^[37]^. The IFN-γ pathway plays an important regulatory role at the center stage of primary, adaptive and acquired resistance to ICB^[37]^. IFN-γ, which is secreted primarily by T cells and NK cells, is crucial to initiate an effective antitumor immune response as it mediates increased MHC-I expression, recruitment of immune cells and direct antiproliferative and pro-apoptotic effects in cancer cells^[129]^. However, constitutive IFN-γ exposure might initiate immunoediting and subsequent immune escape by promoting alteration of the molecules involved in IFN signaling pathways such as Janus kinase/signal transducers and activators of transcription (JAK/STAT) and induction of EMT^[37]^. For example, an increased frequency of IFN-γ pathway gene mutations in tumors of melanoma patients that did not respond to the anti-CTLA-4 antibody ipilimumab has been shown^[37,138]^. Mutations in the IFN-γ pathway could also result in lack of IFN-γ-induced PD-L1 expression causing primary resistance to PD-1 blockade^[37,139]^.

Cancer cell activation of the Wnt/β-catenin pathway or activation of the phosphoinositide 3-kinase (PI3K)/AKT signaling pathway, through, e.g., acquired loss of the tumor suppressor protein phosphatase PTEN, have both been shown to regulate EMT, and both signaling pathways have been mechanistically linked to immune resistance in preclinical studies^[140-144]^. Trujillo and colleagues performed a molecular analysis of baseline and treatment-resistant tumor samples from two malignant melanoma patients who initially showed a durable partial response to either a melanoma-peptide/interleukin-12 vaccine or combined anti-CTLA-4 + anti-PD-1 therapy and subsequently developed new treatment-resistant metastases. Transcriptional profiling and genomic sequencing for oncogenic alterations as well as histologic analysis for T cell infiltration were performed to investigate mechanisms of acquired resistance to immunotherapy^[144]^. In the first case, the authors found increased tumor cell intrinsic activation of β-catenin in the patient’s treatment-resistant metastasis, whereas in the second case genomic sequencing revealed acquired biallelic PTEN loss, while both cases were associated with loss of T cell infiltration^[144]^. This study highlights that continued analysis of acquired resistance samples is crucial to identify potentially targetable resistance pathways amenable to therapeutic intervention.

Expression of IL8 by tumor cells has been shown to promote their EMP^[108]^. Thus, IL8 and its receptor CXCR1/2 axis have been established as potential EMP regulating factors and subsequent targeting of the IL8/CXCR1/2 axis has been explored as a promising EMP target. Preclinical studies have also been performed combining CXCR1/2 blockade with bifunctional anti-TGF-β RII/PD-L1^[145]^. In this study, Horn and colleagues were able to show that simultaneous inhibition of TGF-β, PD-L1 and CXCR1/2 in murine breast and lung cancers synergized to reverse EMP and associated mesenchymal features, reduce infiltration by immunosuppressive MDSCs, and enhance T cell infiltration and overall antitumor immune response^[145]^.

### Clinical trials: targeting EMP to improve immunotherapy of cancer

Combination therapies being tested that seek to enhance the immune response have been extensively reviewed elsewhere^[49,146]^. From the discussion above, it is evident that it is not clear which combination of the multifaceted characteristics of EMP drives the prominent resistance to ICB, and thus it is also not clear how EMP may be targeted most effectively in various cancer contexts. The multiple pathways involved in the initiation and maintenance of epithelial plasticity and the redundancy between these overlapping pathways makes EMP a challenging target. Attempts are being made to target EMP through the multitude of soluble factors, receptors, and transcription factors involved. Several combinations with ICB have been explored in preclinical models, and a few trials have been initiated based on the hypothesis that EMP is one of the main mediators compromising the efficacy of ICB therapies and that targeting EMP could lead to stronger and more durable ICB responses [Table 1]. Here, we will specifically highlight promising therapeutic agents targeting EMP-mediated mechanisms of ICB resistance and discuss them in light of the available mechanistic data to support their multifaceted mechanism of action within the TIME.

**Table 1:** Clinical trials where EMP targets are being evaluated in combination with ICB targeting the PD-1/PD-L1 axis

Target	Drug name	Type	Cancer type	Latest phase^*^	Clinical trial number^**^
TGF-βRI	Vactosertib + durvalumab	Selective TKI	Urothelial	II	NCT04064190
TGF-βRI	LY3200882	Selective TKI	Advanced cancer	II	NCT04158700
PD-L1/TGF-βRII	Bintrafusp-alfa	AntiPD-L1/TGFbetaRII fusion protein	NSCLC	III	NCT03631706
			Biliary tract	III	NCT04066491
			Cervical	II	NCT04246489
(TYRO3, AXL, MER)/KIT/VEGFR2	Sitravatinib	Pan-TKI	NSCLC	III	NCT03906071
			ccRCC	II	NCT03680521
			Urothelial	II	NCT03606174
c-MET/VEGFR2/AXL/RET	Cabozantinib	Pan-TKI	RCC	III	NCT03937219
AXL	Bemcentinib	Selective TKI	NSCLC	II	NCT03184571
			Mesothelioma	II	NCT03654833
			Breast cancer	II	NCT03184558
			Melanoma	II	NCT02872259

^*^Only phase II or later trials are shown. Trials designated as phase I/II are listed as latest phase = II; ^**^Trial identifiers and associated information obtained from www.clinicaltrials.gov. TKIs: tyrosine kinase inhibitors; NSCLC: non-small cell lung cancer; RCC: renal cell carcinoma; TGF-β: transforming growth factor-beta; PD-L1: programmed death-ligand 1

TGF-β is a major regulator of the tumor microenvironment, and also a potent inducer of cancer cell EMT and immune suppression^[61,86,97,98]^. TGF-β is being explored as a target for combination with ICB. Recent retrospective and preclinical reports highlight its role in ICB resistance, while mechanistic and clinical studies continue to refine targeting strategies and synergy with checkpoint blockade. TGF-β is expressed in cancer, stromal and immune cells in three isoforms, TGF-β1, β2, and β3. Several pan-cancer gene expression analyses have shown that TGF-β1 is the primary isoform expressed in tumors, while TGF-β3 is less frequently observed and TGF-β2 is rarely detected and mainly serves to regulate normal cardiac function and hematopoiesis, which helps explain the failure of earlier pan-TGF-β targeting therapies due to toxicity-related adverse events^[108,147,148]^. The most common proposed mechanisms of TGF-β-driven ICB resistance are CD8+ T cell exclusion via fibroblast differentiation and subsequent stromal remodeling and inhibition of CD4+ T helper cell differentiation to the immune-activating T-helper (Th)1 effector cell phenotype, as these conditions have been reproduced in various mouse tumor models and effectively treated by combined targeting of TGF-β and the PD-1/PD-L1 axis^[149-152]^. Additionally, anti-TGF-β was shown to act in synergy with ICB by suppressing immunosuppressive Tregs and EMT in cancer cells in a murine squamous cell carcinoma (SCC) model^[153]^. However, not all tumor models respond equally well to the combination since its efficacy seems to be highly dependent on CTL anti-tumor activity, suggesting that the most immunogenic tumors are expected to benefit the most from this combination^[150]^.

TGF-β-targeting therapies in combination clinical trials with ICB for advanced solid cancers include the small molecule tyrosine kinase inhibitors (TKIs) vactosertib and LY3200882, which specifically target TGF-βRI signaling via ALK5 inhibition (NCT04158700, NCT04064190, NCT02937272)^[108]^. The most advanced of these, galunisertib, has been shown to inhibit *in vivo* cancer cell invasion and metastasis in addition to the mechanisms mentioned above^[154]^; however, it also inhibits p38α due to its similarity to ALK5, and this may contribute to potentially serious side effects that have caused other TKIs in this class of drugs to fail^[155]^. It was recently announced that Eli Lilly discontinued the development of galusertinib. However, several alternative TGF-β-targeting strategies are also being tested in the clinic, including AVID200, a novel fusion protein that specifically traps free TGF-β1/3 and has recently shown promising safety in a phase I monotherapy trial^[148]^, and bintrafusp-alfa (formerly M7824), an anti-PD-L1/TGF-βRII fusion protein that functions both as a checkpoint inhibitor and a TIME-localized TGF-β trap. Bintrafusp alfa has shown superior efficacy in several murine models compared to anti-PD-L1 and anti-TGF-β administered both alone and in combination while also reversing TGF-β1-induced tumor cell plasticity^[108]^, prompting multiple clinical trials in various malignancies including lung (NSCLC and SCLC), breast, cervical, and colorectal (CRC) cancers as monotherapy or in combination with radio- or chemotherapy (NCT02517398, NCT03554473, NCT03620201, NCT03524170, NCT03579472, NCT04246489, NCT03436563). Notable among these is the trial in second-line advanced NSCLC (unselected for PD-L1 expression level), which has shown durable responses and encouraging long-term survival while maintaining a manageable safety profile over a two-year follow-up period, prompting a randomized phase III clinical trial *vs*. pembrolizumab in first-line PD-L1-high NSCLC (NCT03631706)^[156]^.

AXL receptor tyrosine kinase (RTK) has been consistently linked to EMT-mediated drug resistance in a number of cancers^[157]^, and thus represents a promising drug target in this context. AXL expression is prevalent in EMT- and stem cell-related gene expression profiles, highlighting its role in facilitating plasticity phenotypes^[95,158-160]^. AXL signaling has also been shown to contribute uniquely to both tumor intrinsic and microenvironmental immunosuppression and is initiated by its ligand Gas6 as well as heterodimerization with other RTKs. Antony and colleagues showed that GAS6-AXL signaling network is a mesenchymal molecular subtype-specific therapeutic target in ovarian cancer^[161]^. Of note, this paper also elegantly demonstrated the crucial role of AXL moving from cholesterol-rich lipid rafts in epithelial carcinoma cells to the normal plasma membrane domain where it multimerizes with other RTKs in mesenchymal carcinoma cells^[161]^. In a later study, Antony and co-authors were able to demonstrate that the tumor suppressor opioid-binding protein/cell adhesion molecule (OPCML), which is silenced in over 83% of ovarian carcinomas by loss of heterozygosity or by epigenetic mechanisms, is spatially restricted to the cholesterol-rich lipid rafts together with AXL, and when present, represses AXL-dependent oncogenic signaling through protein tyrosine phosphatase receptor type G (PTPRG) in a coordinated manner^[162]^. This study also serves to illustrate how two spatially restricted tumor suppressors, OPCML and PTPRG, coordinate to repress AXL-dependent oncogenic signaling in the Epi context, and where the loss of lipid raft organization upon EMT could lead to overactivation of AXL and more promiscuous heterodimerization with other RTKs in the M state. Thus, a therapeutic strategy to re-introduce OPCML represents an opportunity to target signaling networks, as opposed to more linear systems^[158]^. AXL signaling activates downstream MAPK/ERK and PI3K/Akt pathways, which are known to drive cell survival, proliferation, migration and invasion, both of which are crucial for cancer progression and associated with poor patient outcomes^[157,163]^. AXL inhibition in malignant tumors holds the potential to target AXL-mediated epithelial plasticity, stimulate antigen presentation, and block the recruitment of immunosuppressive macrophages to the TIME^[164,165]^. As such, several AXL-targeting therapeutics have emerged and are in various phases of clinical development in combination with ICB^[108]^. Although not initially developed as AXL-targeting agents, pan-TKIs that inhibit a spectrum of RTKs including AXL, most notably cabozantinib and sitravatinib, have shown promising activity in combination with ICB particularly in ICB-naïve renal cell carcinoma (NCT03937219, NCT03680521). The most advanced clinical stage selective small molecule AXL kinase inhibitor is bemcentinib (BGB324, formerly R428)^[159,166]^. Bemcentinib is currently in phase II development for a variety of malignancies including NSCLC (NCT03184571), mesothelioma (NCT03654833), triple-negative breast cancer (NCT03184558), and melanoma (NCT02872259) in combination with pembrolizumab. A recent presentation of results from an ICB-naive, advanced NSCLC cohort treated with bemcentinib and pembrolizumab (NCT03184571) reported improved overall response and disease control rates with a promising safety profile compared to previous benchmarks of pembrolizumab monotherapy and salvage chemotherapy. Strikingly, transcriptional analysis showed that pretreatment tumor biopsy samples from patients who turned out to be durable responders to the combination treatment displayed significant upregulation of EMT and myeloid activation gene expression signatures including *AXL* and *TGFB1*. In contrast, a negative regulation of EMT gene signature was downregulated; notably, *PD-L1* and *INFG* levels were not associated with response. Importantly, over half the patients in the cohort were also PD-L1 negative (< 1% tumor positive cells) as assessed by IHC staining of pretreatment biopsies, although the particular assay does not account for PD-L1 expression in immune cells which may play a significant role^[167]^. By the same token, stratification of patients based on AXL IHC staining of both tumor and immune cells was associated with significantly improved patient survival^[168]^. In addition to enhancing responses to non-inflamed (PD-L1-low) tumors, EMT targeting is currently being pursued as a strategy to restore the efficacy of ICB by rechallenging ICB-refractory patients with EMT-ICB combination therapies. In NSCLC, ICB-refractory patients are increasingly treated with salvage chemotherapy, as prior ICB regimens appear to have a chemo-sensitization effect^[169]^. However, reversing the acquired ICB resistance may have an even more desirable effect. Phase II ICB combination trials of both bemcentinib (NCT03184571) and sitravatinib (NCT02954991) in ICB-refractory NSCLC have shown promising results compared to salvage chemotherapy benchmarks, and have allowed the advancement of sitravatinib to a phase III randomized trial *vs*. docetaxel (NCT03906071) and also to a phase II trial in ICB-refractory urothelial carcinoma (NCT03606174).

Members of the TAM family of RTKs (TYRO3, AXL and MER) have emerged as attractive targets for cancer therapy, and they have received a great deal of attention for ICB combination due to their suppressive functions in innate immune cells^[19,170]^. Thus, TAM receptor inhibition is expected to unleash an innate-driven antitumor immune response; however, blocking all three family members is expected to yield intolerable side-effects. Thus, specific inhibitors targeting one family member only are most advanced in clinical testing. Selectively targeting only MERTK is also expected to have ambiguous effects; in addition to expression on a myriad innate immune cells MERTK has also emerged as a T-cell costimulatory molecule^[170]^. Thus, targeting MERTK in the TIME is expected to negatively affect T cell functionality, rendering PD-1 therapy counter-productive^[170]^. Targeting the other family members AXL or TYRO3 with specific inhibitors is expected to yield a primarily antitumor immunological effect as these receptors predominantly function as immune-inhibitory receptors on APCs.

Accordingly, it is important to stress that targeting a given pathway may affect several of the innate or acquired resistance mechanisms mentioned in the previous sections due to the concerted upregulation of the pathway in multiple compartments of the TIME and the diverse yet coordinated functions that this conserved biological program engages; thus, the observed effect when combining targeted therapies with ICB in a given patient population may also be driven by one or more uncharacterized mechanisms of action. Using AXL targeting as an example, apart from its expression on subpopulations of mesenchymal cancer cells, AXL is also present on a number of immune cell types, particularly myeloid-derived suppressor cells and M2 polarized macrophages, where it confers an immunosuppressive role in tumor and normal inflammatory environments^[163,170]^. AXL expressing DCs were shown to display phenotypic and functional diversity, and this population of cells was suggested to represent *in situ* plasticity of the plasmacytoid dendritic cell lineage^[171]^. Recently, an AXL+ regulatory DC population that strongly suppresses antitumor immunity was characterized^[172]^. AXL is a well-established negative-feedback regulator of the innate immune response by dampening the TLR response^[173]^, and as AXL inhibition has been shown to elicit an immunogenic form of cell death in cancer cells, it is expected to initiate a strong innate anticancer immune response through recruitment and activation of APCs^[53,124,170]^. Furthermore, inhibiting AXL has significant anti-angiogenic potential which may normalize the tumor vasculature, reduce hypoxia and play a role in reversing ICB resistance^[146]^; not only does AXL signaling blockade in tumor cells decrease secretion of pro-angiogenic factors, but it also directly impairs vascular endothelial growth factor A-dependent angiogenesis in vessel endothelial cells, which ubiquitously express AXL^[165]^.

## Conclusion

From *in vitro* models and preclinical *in vivo* models, targeting EMP has emerged as one of the most promising strategies to increase both the response rates and duration of responses to ICB. Since most novel EMP-targeting drugs are currently in early phases of clinical development, it follows that these therapies must first be tested on patients who have failed currently approved standard-of-care treatments, which in many cases include ICB. Reversal, rather than prevention, of EMP is therefore being tested in most current clinical trials [Table 1], although for most compounds, the optimal strategy would be to move from second-line combination testing to 1st line combination with ICB. In the long term, based on preclinical evidence that EMP targeting might also enhance natural cancer immunosurveillance in a cancer preventive setting, it would be of great interest to also evaluate the efficacy of several drug candidates mentioned above in Table 1 in chemoprevention. In the chemopreventive context specifically, it will be important to monitor potentially toxic long-term effects, as the targeted pathways are expected to also affect both the immune and stem/progenitor cell populations necessary for maintaining physiological homeostasis and repair processes^[78]^.

Most of the genomic studies thus far have relied solely on analyses of pretreatment samples, and thus the results obtained provide a brief snapshot that may be useful to dissect the hallmarks of innate resistance *vs*. response, whereas longitudinal sampling would provide deeper insight into the cellular heterogeneity and dynamics of acquired therapy resistance and associated intercellular communication. This information is crucial to determine which targets of EMP modulators induce optimal antitumor responses in combination with ICB in a particular context. The sum of the alterations induced in the variety of cellular populations in the TIME eventually constitutes the clinical benefit of these combination regimens. Several clinical scenarios as explained above could benefit from a rational combination strategy including EMP inhibitors [Table 1], and biomarker- or biosignature-driven clinical trial design is also expected to contribute more effectively to our biological understanding. Clearly, targeting EMP impacts the dynamic and fine-tuned regulation of the myriad cellular compartments differently. Although not covered in depth in this review, phenotypic and functional plasticity of immune cells are also expected to be affected by interventions targeting cancer EMP and contribute to clinical benefit. Much is yet to be learned regarding how EMP targeting might shift the polarization of immune cell phenotypes in a manner that either supports or represses antitumor immunity. High-dimensional single cell technologies are expected to shed light on these important questions regarding the spatial and temporal regulation of EMP in the TIME. The complex intercellular communication within the TIME, although much remains unknown, holds significant promise; increased knowledge regarding this intricate interplay may enable the coordinated polarization of cancer and immune cells in the TIME to be effectively targeted through EMP in a tailored and therapeutic fashion to improve the efficacy of cancer ICB combination therapies.

## Declarations

### Copyright

© The Author(s) 2020.
